# Use of three-dimensional–three-dimensional digital subtraction angiography spin fusion protocol for complex aortoiliac endovascular interventions

**DOI:** 10.1016/j.jvscit.2021.03.008

**Published:** 2021-04-20

**Authors:** Edvard Skripochnik, Nicole D'Ambrosio, William E. Crawford, Shang A. Loh

**Affiliations:** Division of Vascular and Endovascular Surgery, Department of Surgery, Stony Brook University Medical Center, Health Sciences Center T19-090, Stony Brook Medicine, Stony Brook, NY

**Keywords:** Complex aortic anatomy, Endovascular aortic repair, Fusion imaging, Siemens

## Abstract

Complex endovascular aortic interventions in patients with excessive tortuosity or difficult gantry angles can be challenging. Although fusion imaging can help navigate these issues, it is based on preoperative imaging studies, which becomes skewed after introduction of stiff wires and large devices into the aorta. The subtraction spin protocol performs two cone-beam computed tomography scans to create a subtracted image of the contrast-filled vessels after wire and device placement to accommodate vessel distortion. We have reported a complex fenestrated endovascular aneurysm repair case with a highly angulated neck to highlight the advantages of the subtraction spin protocol in anatomically hostile endovascular repairs.

Fusion imaging has been adopted as a useful supplement during endovascular aneurysm repair (EVAR) to speed vessel cannulation and device placement and reduce the operative time, contrast load, radiation dose, and blood loss.^1^, ^2^, ^3^, ^4^ However, the current fusion technology relies on preoperative computed tomography (CT) angiography (CTA), which can be problematic owing to suboptimal imaging quality, poor contrast timing, patient positioning, and anatomic changes over time from CTA to surgery. More importantly, on insertion of stiff wires, sheaths, and devices, the degree of anatomic change to the branch vessels and lengths compared with the preoperative imaging findings will be underestimated. In the present report, we have described a subtraction spin protocol to facilitate a complex endovascular aortic repair with difficult anatomy.

## Methods

### Subtraction spin protocol

**Fig 1:**
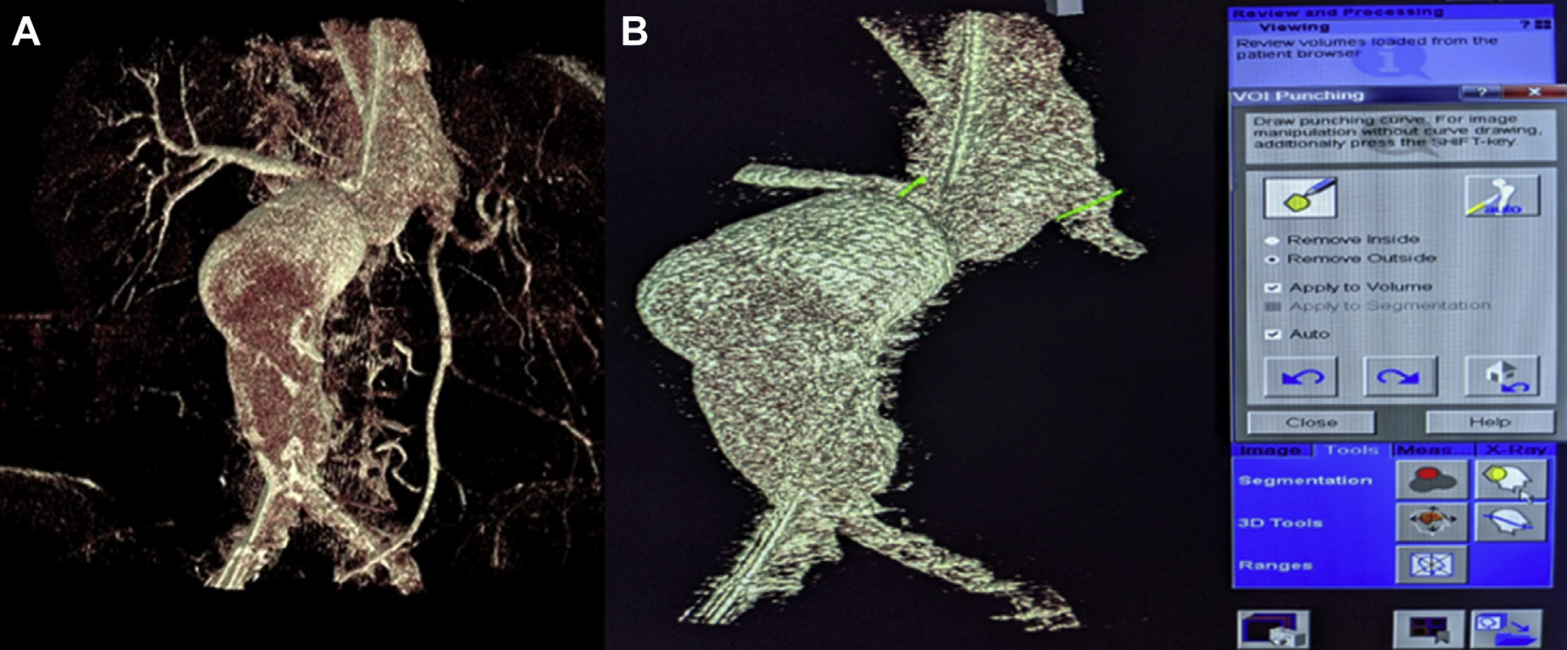
**A,** Three-dimensional (3D) reconstruction from the DynaDSA spin. **B,** Volume of interest punch tool is selected from the tools dropdown menu to remove all distracting anatomy.

**Fig 2:**
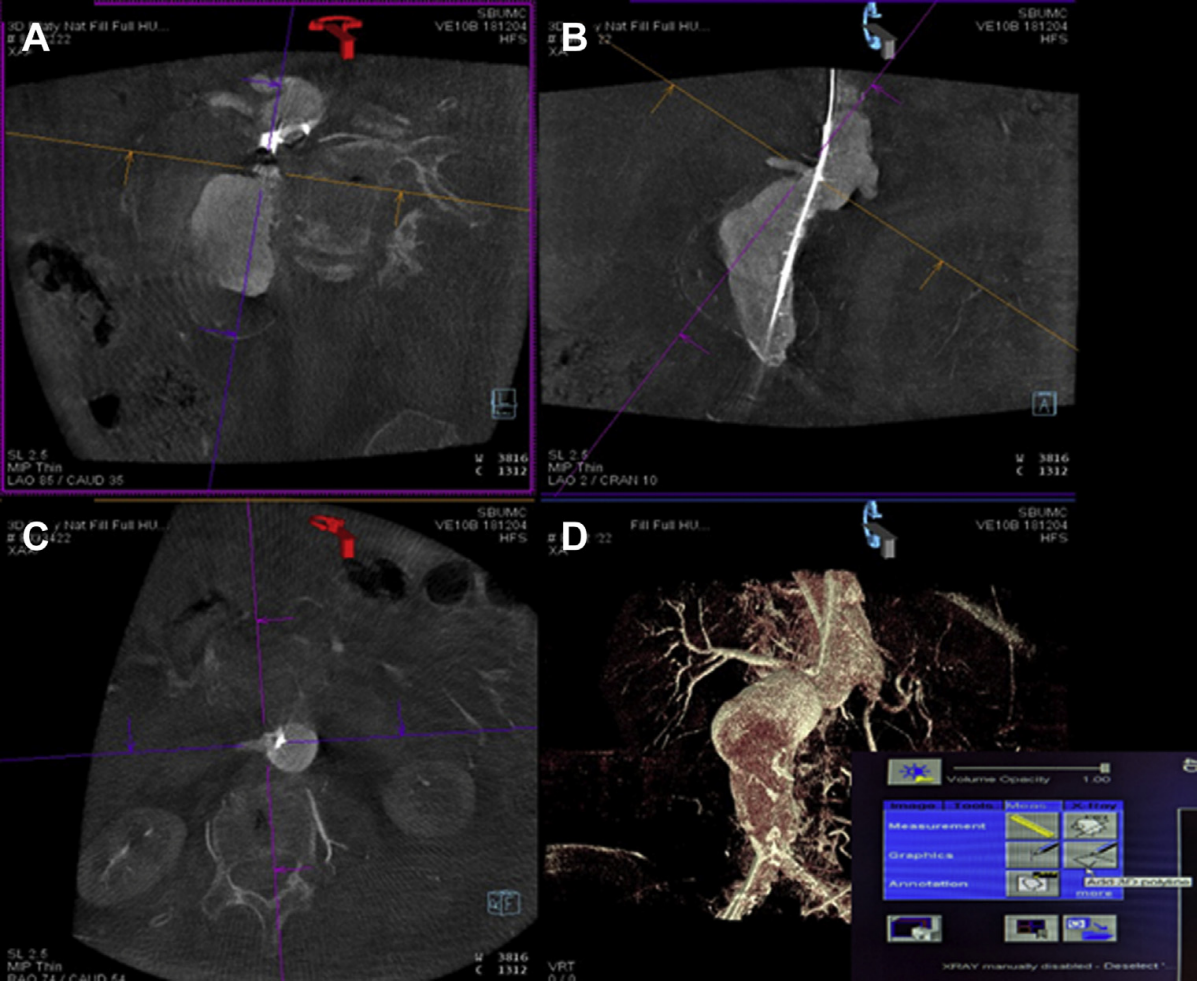
Digital subtraction angiography (DSA) spin multiplanar reformation planning for overlay using sagittal **(A)**, coronal **(B)**, and axial **(C)** views. This screen is also used to center on each visceral vessel and the polyline tool **(D)**, selected from the Mods tab, is used to outline each vessel of choice one at a time.

**Fig 3:**
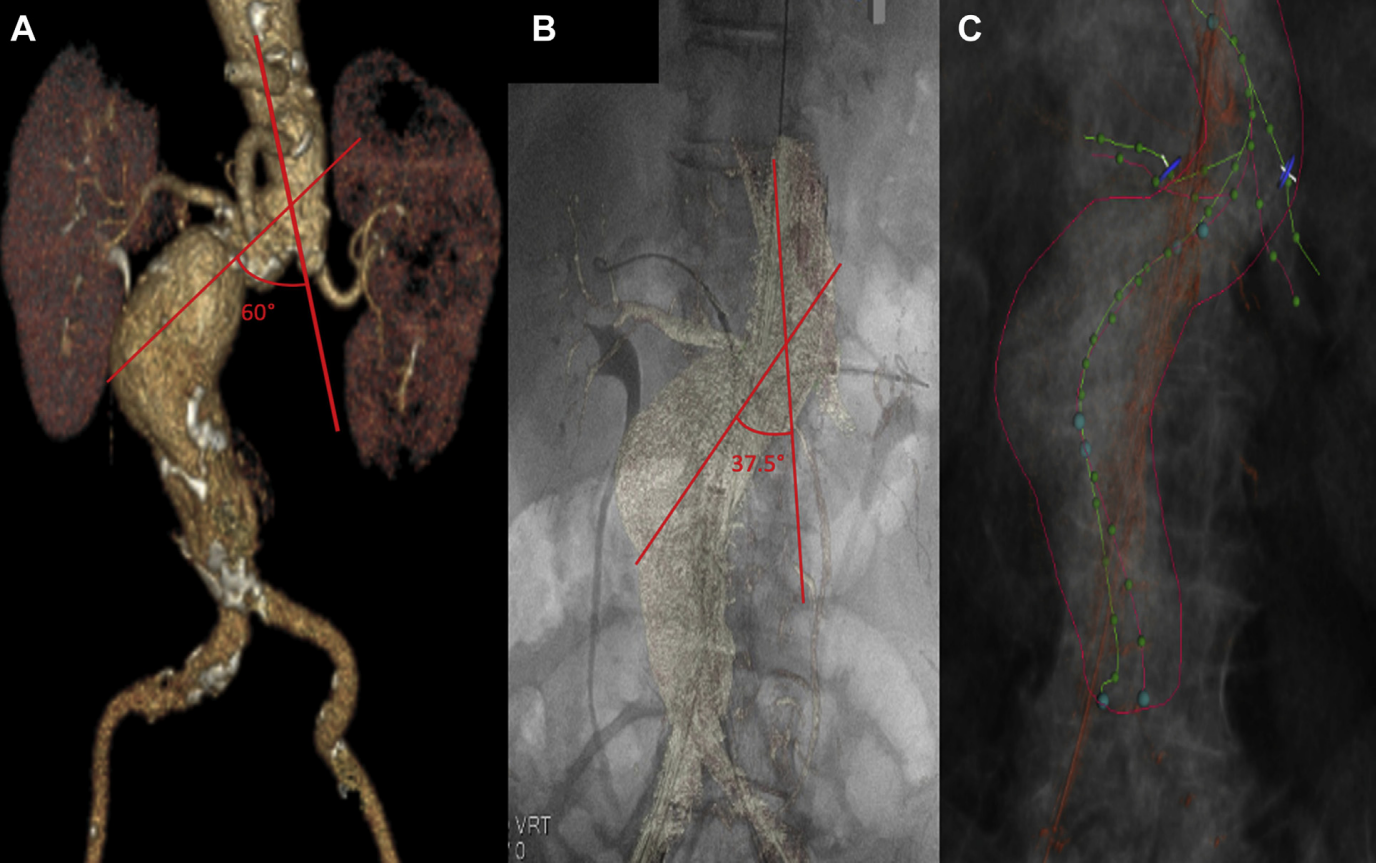
A, Three-dimensional (3D) reconstruction of the preoperative computed tomography (CT) angiogram (CTA). As denoted by the *red lines,* a 60° angle is present at the level of the renal arteries. **B,** After device introduction and the subtraction spin protocol, we observed a 37.5° angle at the level of the renal arteries. **C,** Polylines are drawn for the fusion from the preoperative computed tomography (CT) scan (*red polyline*) and from the subtraction spin (*green lines*) to show the differences in the aortic and renal configuration before and after device deployment.

After catheter and stiff wire and/or device placement, the 4s digital subtraction angiography (DSA) acquisition protocol is selected. After appropriate positioning, the Siemens DynaDSA protocol (Siemens Medical Systems, Erlangen, Germany) performs the initial DSA mask spin on a Siemens ARTIS pheno machine (Siemens Healthineers, Erlangen, Germany). After the first masking rotation is completed, a second CT spin with a 15-mL/s for a 90-mL half-strength contrast injection is completed using a Medrad Arterion 7 power injector (Bayer HealthCare LLC, Whippany, NJ) with an x-ray delay of 1 second. An interactive three-dimensional (3D) reconstruction is performed based on the acquired volume rendering technique (VRT) using the VRT vessels preset, sharp image characteristics, and manual volume of interest size (Fig 1, *A*). Volume rendering can be used to reveal vessel abnormalities by allowing for direct visualization of the scanned volume data.^2^ Using the volume of interest punch tool, the surrounding distracting anatomy is removed to allow for better visualization of the relevant anatomy (Fig 1, *B*). Next, vessel planning is performed using the three multiplanar reformations, which is the method used to convert between planes within a given imaging modality (Fig 2). One by one, each vessel is centered on all the multiplanar reformations, and a 3D polyline is drawn on the sagittal outline of the ostia of interest (Fig 2). The accuracy of the polyline is confirmed on the VRT image. The 3D objects are then overlaid on the live 3D fluoroscopy image. EVAR guidance can then be used to map the contour and centerline of the aorta and vessels of interest. Minor manual adjustments can then be made as necessary based on a limited aortogram. The EVAR guidance allows for the ability to measure the degree of anatomic changes after the introduction of the stiff wires and sheaths (Fig 3).

### Case report

A 70-year-old woman with chronic obstructive pulmonary disease and a hostile abdomen had presented with an asymptomatic 6.5-cm juxtarenal abdominal aortic aneurysm with a 7-mm neck. Open repair was a poor option given her history of open colon resection with subsequent hernia and mesh complications. Preoperative CTA demonstrated an 80° angle at the infrarenal neck and an 80° angle in the opposite direction at the suprarenal aorta. The right and left renal arteries both emerged at a 45° angle relative to the neck and both were downward going. A Cook Zenith Fenestrated graft (Cook Medical, Bloomington, Ind) with two small fenestrations and a scallop was built using a modified centerline to account for the anticipated graft position after insertion. The insertion of Lunderquist wires bilaterally significantly changed the working angles. Using the subtraction spin protocol, the superior mesenteric and renal artery origins were redefined for the fusion overlay. Significant angle variation was observed at the aortic neck and renal ostia from an ∼60° to 37.5° angle (Fig 3). The gantry angles needed to achieve a perpendicular view of the renal origins according to the new fusion markers also changed significantly from the preoperative measurements by 13° to 20°. After vessel marking and mapping, the Cook fenestrated proximal piece was inserted, and a limited angiogram of the visceral segment revealed marker accuracy; thus, no adjustments were necessary. This significantly aided in graft positioning by facilitating placement slightly superiorly to assist in cannulation of the severe downward going left renal artery and avoiding the need for any additional angiograms through cannulation. The total fluoroscopy time was 80.5 minutes, the total fluoroscopy dose was 3.6 Gy, and the total contrast was 101 mL for this case. At the last follow-up, the patient was >1 year after treatment without evidence of an endoleak and with patent renal artery stents and aneurysm sac shrinkage.

## Discussion

Evolving technologies have continued to allow endovascular specialists to expand the boundaries of aortic surgery. Fusion of preoperative CTA with intraoperative two-dimensional or 3D imaging can reduce the cannulation time and, therefore, reduce the radiation dose, contrast amount, and time required.^1^^,^^2^^,^^5^ However, the steep gantry angles necessary to visualize the renal and visceral vessels in a complex aortic case, compounded by a large body mass index, will exponentially increase the radiation amount to both patient and operator.^1^^,^^6^^,^^7^ Changes to the vasculature from the time of scanning to surgery and, most importantly, after the insertion of devices, have led some operators to use adjuncts such as intravascular ultrasound or multiple DSA runs with manually adjusted overlays. However, these are limited by the additional operative time, wire manipulation, cost, and learning curve required.^2^

Using subtraction spin requires a 90-mL injection at half strength contrast and delivers ∼280 mGy, which can be expected for cases of difficult visceral cannulation and accounts for ∼10% of the radiation required during a complex aortic case (2-3 Gy). Additionally, it eliminates the requirement for highly angulated pictures through large body mass territories for visceral cannulation. Most importantly, the altered vessel configuration when performing fenestrated EVAR or branched EVAR means accurate building of a custom centerline preoperatively is crucial to estimating the position of the fenestrations.

The subtraction spin protocol is an underused tool that can mitigate the limitations of traditional fusion imaging for complex anatomy. Performing the protocol after the insertion of stiff wires or devices allows the operator to adjust the gate orientation or fenestration alignment to the accurate markers. The use of this technique highlighted the degree of movement of the aorta and the renal artery orientation in the present patient with a highly angulated aortic neck. The fluoroscopy time was increased, and the air kerma was ≤3.6 Gy, within the range for complex aortic work of 2.5 to 5 Gy.^1^^,^^7^, ^8^, ^9^ Some have questioned the additional radiation and contrast load required for a subtraction spin. However, in complex cases, these concerns will be leveled by the reduction in the DSA runs and overall fluoroscopy time.

Case planning and device deployment in tortuous and highly angled anatomy remain an Achilles heel of endovascular aortoiliac stent grafting. Until real-time adjustments can be performed according to the type of devices inserted and associated conformation changes, the subtraction spin protocol offers an accurate and low risk solution to assist intraoperative navigation.

Although subtraction spin imaging has helped with difficult cases of EVAR, it is difficult to generalize given the need to train personnel, the requirement for increased dual cone-beam CT (CBCT) radiation and contrast doses, and the additional confirmation changes with the use of a stiffer device, which pose limitations. However, widespread baseline fusion imaging training has made the addition of subtraction spin protocol training more feasible. Although dual CBCT delivers additional radiation and contrast doses, this is likely compensatory given that multiple DSA runs would be required in its stead. Finally, inaccuracies in the fusion markers can be reduced by inserting the endograft before the CBCT. However, this could increase scatter in the CBCT and potentially obscure the vessels of interest.

## Conclusions

The use of the subtraction spin protocol can mitigate many of the disadvantages of standard fusion imaging. The case we have presented has shown the applicability, accuracy, and ease of the use of subtraction spin guidance in complex aortic cases with severe angulation or difficult gantry angles.
